# The development of epiretinal membrane following rhegmatogenous retinal detachment repair: incidence, risk factors, and outcomes

**DOI:** 10.5935/0004-2749.20220032

**Published:** 2025-08-21

**Authors:** Yan Fu, Tian-hao Xie, Zhao-hui Gu, Li-ying Li, Qiang Chen, Yue-ling Zhang

**Affiliations:** 1 Department of Ophthalmology, Baoding No.1 Central Hospital, Baoding 071000, Hebei Province, China; 2 Affiliated Hospital of Hebei University, Baoding 071002, Hebei Province, China

**Keywords:** Epiretinal membrane, Retinal detachment, Scleral buckling, Visual acuity, Vitrectomy, Membrana epirretiniana, Descolamento da retina, Recurvamento da esclera, Acuidade visual, Vitrectomia

## Abstract

**Purpose:**

To investigate the incidence, risk factors, and visual outcomes of epiretinal membrane development following rhegmatogenous retinal detachment repair.

**Methods:**

This was a retrospective study of 309 eyes that underwent initial surgery for primary uncomplicated rhegmatogenous retinal detachment. Examinations were conducted preoperatively and then postoperatively at 1, 3, 6, and 12 months. The study patients were categorized into two groups depending on the presence or absence of the epiretinal membrane.

**Results:**

The incidence of postoperative epiretinal membrane was 28.5%; 42.7% of these patients had severe epiretinal membrane development and therefore underwent the epiretinal membrane removal. Logistic regression analyses revealed that giant retinal tears (OR: 2.66; 95% CI: 1.045-6.792, p=0.040) and horseshoe tears (OR: 0.534; 95% CI: 0.295-0.967, p=0.039) were the significant predictors of postoperative epiretinal membrane. Triamcinolone acetonide staining was significantly associated with the prevention of epiretinal membrane (p=0.022). A total of 34 patients showed a better or an equal final best-corrected visual acuity; of which 4 eyes were evaluated at the final follow-up visit and exhibited a reduced best-corrected visual acuity.

**Conclusion:**

Our analysis demonstrated that horseshoe tears and giant retinal tears represent the risk factors for the postoperative epiretinal membrane. Triamcinolone acetonide staining had a significant preventive effect on the postoperative epiretinal membrane. Furthermore, a second round of pars plana vitrectomy, including membrane removal, led to a significant improvement in the final best-corrected visual acuity as per the last follow-up examination, albeit the recovery was limited.

## Abstract (pt)

**Objetivos:**

Investigar a incidência, fatores de risco e desfechos visuais do desenvolvimento da membrana epirretiniana após reparo do descolamento regmatogênico da retina.

**Métodos:**

Trata-se de um estudo retrospectivo de 309 olhos submetidos à cirurgia inicial para descolamento regmatogênico da retina primário sem complicações. Os exames foram realizados no pré-operatório aos 1, 3, 6 e 12 meses pós-operatórios. Os pacientes foram divididos em dois grupos, dependendo da presença ou ausência de membrana epirretiniana.

**Resultados:**

A incidência de membrana epirretiniana pós-operatória foi de 28,5%; 42,7% desses pacientes apresentaram desenvolvimento grave da membrana epirretiniana e, portanto, foram submetidos à remoção desta membrana. A regressão logística mostrou que as lágrimas retinianas gigantes (RC: 2,66; 95% IC: 1,045 - 6,792, p=0,040) e lágrimas em ferradura (RC: 0,534; 95% IC: 0,2950,967, p=0,039), foram preditores significativos de membrana epirretiniana pós-operatória. A coloração com acetonida de triancinolona foi significativamente associada à prevenção da membrana epirretiniana (p=0,022). Trinta e quatro pacientes apresentaram acuidade visual melhorada, ou igual, ou acuidade visual final melhor corrigida; 4 olhos foram avaliados na consulta final de acompanhamento e apresentaram redução da acuidade visual melhor corrigida.

**Conclusão:**

Nossa análise demonstra que as lágrimas de ferradura e as lágrimas retinianas gigantes representam fatores de risco para a membrana epirretiniana pós-operatória. A coloração com acetonida de triancinolona teve um efeito preventivo significativo na membrana epirretiniana no pós-operatório. Além disso, uma segunda rodada de vitrectomia pars plana, incluindo remoção da membrana, levou a uma melhora significativa da acuidade visual final melhor corrigida na última consulta de acompanhamento, embora a recuperação tenha sido limitada.

## INTRODUCTION

The development of the epiretinal membrane (ERM) following rhegmatogenous retinal detachment (RRD) is a relatively common complication that can lead to reduced vision acuity, stereopsis, and metamorphopsia(1). The incidence of ERM formation after RRD repair has been reported to be 3%-51.1%^(2-6)^. The development of severe ERM can also cause contraction and distortion of the retina, requiring a second pars plana vitrectomy (PPV) to remove the ERM. In the majority of the cases, a combination of PPV and ERM peeling may restore macular anatomy, improve visual acuity, and alleviate metamorphopsia. However, in some cases, the patients undergoing ERM peeling following RRD surgery do not show any improvement in their visual acuity. Furthermore, there is some degree of uncertainty with regards to the outcomes of patients with postoperative ERM.

Therefore, we conducted a retrospective review of a large cohort of patients who received an initial round of scleral buckling (SB) surgery or PPV for primary uncomplicated RRD. Our specific aims through this review were to (1) determine the incidence of ERM formation after RRD repair and the rate of secondary PPV for ERM peeling, (2) to evaluate the effects of various preoperative and intraoperative factors on the formation or prevention of postoperative ERM, and (3) to investigate the visual outcomes of patients undergoing secondary PPV for ERM peeling.

## METHODS

### Study design

This was a retrospective study involving patients who received an initial SB surgery or PPV for primary uncomplicated RRD. The research was conducted in accordance with the Declaration of Helsinki. A signed informed consent form was obtained from all patients involved in this study for their participation.

This study involved 309 consecutive eyes that were evaluated during January 1t, 2015-2018 at the Department of Ophthalmology, Baoding No.1 Central Hospital, China. Our inclusion criteria for the patients were as follows: (1) a postoperative follow-up period of >12 months, (2) good-quality spectral domain optical coherence tomographic (SD-OCT) scans that could reveal the presence or absence of postoperative ERM development during the follow-up examination, and (3) age of ≥18 years. The exclusion criteria for the study patients were as follows: (1) previous vitreoretinal surgery, (2) redetachment during the follow-up examinations, (3) uveitis, (4) retinal vascular diseases (e.g., diabetic retinopathy or vascular occlusive diseases), (5) retinal dystrophies, (6) preexisting pathological features of the macular (e.g., macular hole, age-related macular degeneration, macular edema, vitreomacular traction syndrome, and ERM), and (7) additional surgery for the postoperative development of proliferative vitreoretinopathy (PVR) or the recurrence of RRD.

All patients were evaluated (for both eyes) preoperatively and at 1, 3, 6, and 12 months after the operation. These evaluations included a complete ophthalmological examination, involving best-corrected visual acuity (BCVA), intraocular pressure (IOP), slit-lamp biomicroscopy, and OCT scans. The subjects were categorized into two groups based on the detection of ERM. We defined postoperative ERM formation as a highly reflective layer on the inner macular surface following RRD surgery, which was identified by the Macular Carl Zeiss Meditec Cirrus HD-OCT 5000.

### Data collection

The following demographic and clinical data were acquired: age; gender; medical and ophthalmic history; previous ophthalmic surgery; high myopia (defined as a preoperative spherical equivalent of ≥-6D); lens status; macular status (on or off, as determined by preoperative OCT); the duration of detachment; extent of detachment; the extent, number, type, and location of breaks; the presence of posterior vitreous detachment (PVD), surgical procedures (SB surgery or PPV), and the presence/absence of postoperative ERM. We also acquired a range of data relating to the surgery depending on whether subretinal fluid drainage and retinotomy was performed; the quadrants of cryopexy; cryopexy time; quadrants of endolaser photocoagulation; the use of triamcinolone acetonide (TA) and perfluorocarbon liquid; and whether surgery was combined with cataract surgery. Postoperative BCVA was determined at each visit for the entire duration of follow-up period. We also employed OCT findings to evaluate the anatomical status of the retina.

### Surgical technique

All surgeries were conducted by 2 experienced retinal surgeons (GZH and ZYL). During SB surgery, we applied cryotherapy to retinal tears and the area of degeneration, followed by using a radially or circumferentially oriented silicone sponge to block the retinal break. During the PPV surgery, we conducted standardized 3-port vitrectomy using a 23- or 25-gauge microincision system. First, we conducted vitrectomy to release the vitreous traction around the retinal breaks and degenerations. We then performed endolaser photocoagulation around the retinal breaks and the areas of degeneration. TA staining was used to visualize vitreous traction and the residual vitreous cortex. Silicone oil was applied to all the eyes because commercialized medical gas was unavailable in the mainland China.

**ERM peeling:** The removal of the ERM by further surgery was only considered when the logarithm of the minimal angle of resolution (logMAR) BCVA was worse than 0.3 or when the patient was experiencing retinal distortion or metamorphopsia.

### OCT assessment

For each patient, we obtained additional serial OCT scans throughout the follow-up period, which enabled evaluation for the presence of ERM. Postoperative OCT parameters included the presence of persistent subretinal fluid (SRF) and cystoid macular edema, along with the integrity of the ellipsoid zone (EZ) and the external limiting membrane (ELM) layer. ‘Damage’ was defined as a disruption or loss of integrity.

### Statistical analysis

All statistical analyses were performed using the Statistical Package for the Social Sciences version 25 (IBM Corp., Armonk, NY, USA). All data were tested for normality prior to the analysis using the Shapiro-Wilk test. Qualitative variables were presented as frequencies and percentages, while quantitative variables were presented as the means and standard deviations. The BCVA data were converted to the logMAR prior to statistical analysis. We determined the logMAR values for counting fingers (logMAR=2.5), hand movements (logMAR=2.7), and light perception (logMAR=3.0). The differences in the categorical data were analyzed using the Chi-squared test, while the unpaired t-test or the Mann-Whitney rank-sum test was used to compare continuous data between the eyes without and with ERM. Paired t-tests were used to compare visual functions between different time points. Multivariate regression analyses were performed to identify potentially confounding parameters. *p*<0.05 was considered to be statistically significant.

## RESULTS

### General characteristics

A total of 367 eyes from 365 patients undergoing the RRD repair were recruited in this study. Of these, 58 patients were subsequently excluded due to preexisting retinal or macular disease (n=25), previous eye trauma (n=7), previous vitreoretinal surgery (n=18), or additional surgeries (n=8). Consequently, 309 patients (309 eyes) were included in the final analysis. One month after RRD repair, OCT imaging revealed an evidence of ERM in 19 of the 309 eyes (6.1%). At 3, 6, and 12 months after the RRD repair, OCT scans identified ERM in 47 (15.2%), 69 (22.3%), and 78 (25.2%) of all cases, respectively. A total of 89 eyes (28.8%) were diagnosed with secondary ERM development during the follow-up period.

### The effects of various perioperative factors on the postoperative formation of ERM

We categorized our patient cohort into 2 groups based on the presence or absence of ERM. Table 1 shows the comparison between these two groups of patients with respect to a range of preoperative characteristics. Univariate analysis identified the presence of horseshoe tears (p=0.003) and giant retinal tears (p=0.013), as significant risk factors for the postoperative formation of ERM. We also performed step-by-step logistic regression analysis to evaluate the effect of confounding variables. Based on our results, giant retinal tears (odds ratio [*OR*]: 2.664; 95% confidence interval [*CI*]: 1.0456.792, p*=*0.040) and horseshoe tears (*OR*: 0.534; 95% *CI:* 0.295-0.96, p=0.039) were the significant predictors for the development of postoperative ERM.

**Table 1:** A comparison of preoperative characteristics and visual outcomes of patient groups with and without ERM

Parameter	Overall (n = 309 eyes)	With ERM (n=89 eyes)	Without ERM (n=220 eyes)	p-value
Sex, n (%)				
Male	166 (53.7)	41 (46.1)	125 (56.8)	0.086^a^
Female	143 (46.3)	48 (53.9)	95 (43.2)	
Age, mean ± SD	47.1 ± 12.8	48.7 ± 13.0	46.5 ± 12.7	0.172^b^
Laterality, n (%)				
Right eye	177 (57.3)	48 (53.9)	130 (59.1)	0.406^a^
Left eye	132 (42.7)	41 (56.1)	90 (40.9)	
Lens status, n (%)				
Phakic	276 (89.3)	81 (91.0)	195 (88.6)	0.540^a^
Pseudophakic	33 (10.7)	8 (9.0)	25 (11.4)	
Detachment duration (days), mean ± SD	13.7 ± 9.6	14.1 ± 7.3	12.3 ± 8.1	0.812^b^
Quadrants of RRD, mean ± SD	2.9 ± 0.73	2.8 ± 0.73	3.0 ± 0.67	0.367^b^
High myopia, n (%)	44 (14.2)	10 (11.2)	34(15.1)	0.337^a^
BCVA (logMAR), mean ± SD				
At baseline	1.09 ± 0.47	1.03 ± 0.58	1.12 ± 0.43	0.149^b^
Final BCVA	0.42 ±0.17	0.47 ± 0.17	0.40 ±0.16	**0.002^b^**
Macular status				
On	108 (35.0)	24 (25.8)	84 (38.2)	0.061^a^
Off	201 (65.0)	65 (74.2)	136 (61.8)	
PVD (%)	218 (70.5)	64 (71.9)	154 (70)	0.059^a^
Giant retinal tears, n (%)	21 (6.8)	11 (12.4)	10(4.5)	**0.013^a^**
Number of breaks, n (%)				
Single breaks	235 (76.1)	68 (76.4)	167 (75.9)	0.926^a^
Multiple breaks	74 (23.9)	21 (23.6)	53 (24.1)	
Type of break, n (%)				
Atrophic holes	108 (35.0)	20 (22.5)	88 (40)	**0.003^a^**
Horseshoe tears	201 (65.0)	69 (77.5)	132 (60)	
Location of breaks				
Superior	188	52	136	0.580^a^
Inferior	121	37	84	
Surgical procedure				
SB	179 (57.9)	46 (51.7)	133 (60.5)	0.157^a^
PPV	130 (42.1)	43 (48.3)	87 (39.5)	

ERM= epiretinal membrane; BCVA= best-corrected visual acuity; logMAR= logarithm of minimal angle of resolution; PVD= poster PPV= pars plana vitrectomy; RRD= rhegmatogenous retinal detachment.

a*P* values according to chi-square tests.

b*P* values according to t-tests.

*P* values that are statistically significant (<0.05) are represented in bold.

Table 2 depicts the comparison of intraoperative characteristics between the groups with and without ERM. These two groups showed similar intraoperative characteristics, except in terms of the use of TA staining, which was significantly associated with the prevention of ERM development (p=0.022).

**Table 2:** Comparisons of intraoperative characteristics between groups of patients with and without ERM

Parameter	Overall	With ERM	Without ERM	p-value
SB, n	179	46	133	
Cryopexy time (seconds)	70.8 ±22.3	73.8 ± 22.7	69.8 ± 22.2	0.292^b^
Quadrants of cryopexy, mean ± SD	1.7 ± 0.9	1.8 ± 0.9	1.7 ± 0.9	0.371^b^
Subretinal fluid drainage, n (%)	54 (30.2)	13 (28.3)	41 (30.8)	0.744^a^
PPV, n	130	43	87	
Perfluorocarbon liquid, n (%)	38 (29.2)	11 (25.6)	27(31.0)	0.520^a^
Quadrants of endolaser photocoagulation, mean ± SD	1.7 ± 0.9	1.8 ± 0.9	1.7 ± 0.9	0.502^b^
Cataract surgery, n (%)	16(12.3)	6(14.0)	10(11.5)	0.688^a^
Retinotomy, n (%)	11 (8.5)	4 (8.7)	7 (5.3)	0.809^a^
TA staining, n (%)	99 (76.2)	38 (88.4)	61 (70.1)	0.022^a^

ERM= epiretinal membrane; SB= scleral buckling; PPV= pars plana vitrectomy; TA= triamcinolone acetonide.

a*P* values according to chi-square tests.

b*P* values according to t-tests.

*P* values that are statistically significant (<0.05) are represented in bold.

### The visual outcomes of patients undergoing a second round of surgery to remove ERM

A total of 38 eyes (42.7%) showed severe secondary ERM development after RRD repair and required another round of surgery for removal. The main characteristics of patients with severe ERM development that required subsequent surgical removal are summarized in table 3. The mean duration of time between the initial RRD repair and the surgical removal of ERM was 10.2 ± 7.2 months. ILM peeling was performed on 32 eyes (84.2%).

**Table 3:** Characteristics of the patients undergoing the removal of ERM secondary to RRD repair

Parameter	
BCVA (logMAR) before ERM removal	0.59 ± 0.15
Type of RRD, n (%)	
Macula-on RRD	15 (39.5)
Macula-off RRD	23 (60.5)
Type of RRD repair surgery, n (%)	
SB	21 (55.3)
PPV	17 (44.7)
Gender, n (%)	
Male	21 (55.3)
Female	17 (44.7)
Age, mean ± SD	49.5 ± 10.6
Lens status, n eyes (%)	
Phakic	33 (86.8)
Pseudophakic	5(13.2)
Duration between RRD repair and ERM remove (months), mean ± SD	10.2 ± 7.2

ERM= epiretinal membrane; BCVA= best-corrected visual acuity; logMAR= logarithm of minimal angle of resolution; RRD= rhegmatogenous retinal detachment.

Preoperative BCVA was 0.59 ± 0.15. Visual acuity improved significantly to 0.41 ± 0.14 at the last follow-up examination after ERM removal (p=0.000). The central foveal thickness after ERM surgery was significantly smaller than the corresponding preoperative values (p=0.002). Our data displays that the number of eyes with disrupted EZ and ELM decreased after surgery (p=0.023). In addition, 34 patients (89.5%) showed a better or equal final BCVA after ERM removal than that after RRD repair. Four eyes (10.5%) showed a reduced BCVA at the final follow-up examination. ERM development resulted in a significant decline of BCVA values at the last follow-up visit when compared with a group of patients without ERM (p=0.002), despite the successful ERM removal (Table 1).

## DISCUSSION

Our analysis revealed that 89 of the patients (28.5%) who underwent SB surgery or PPV for RRD went on to develop ERM. This result concurred well with the findings of previous studies on primary RRD^(2,3)^. We noted that the incidence of ERM after PPV was equal to that after SB. Most postoperative ERM (77.5%) was diagnosed within 6 months of the initial surgery for RRDs. A previous study reported that 92.7% of the patients developed ERM approximately 6 months after the initial vitrectomy^(7)^. In the present study, 38 eyes (42.7%) underwent a second vitrectomy for ERM removal, and 76.3% of these 38 cases underwent a second PPV for ERM removal within 1 year of RRD repair. This result is lower than that reported by previous studies; for example, a past study reported that 77.8% of patients with postoperative ERM required secondary surgery^(8)^. This inconsistency may be related to our rigorous surgical criteria. We performed surgery to remove ERM only when the logMAR BCVA was worse than 0.3, when there was an obvious retinal distortion, or when the patient complained of metamorphopsia.

Previous studies have reported a range of risk factors associated with the formation of ERM after RRD repair, including age^(4)^, equatorial breaks^(8)^, cryopexy^(9)^, repeated operations^(10)^, proliferative vitreoretinopathy^(11)^, macular involvement^(3)^, low preoperative visual acuity^(12)^, and multiple and/or large retinal breaks^(4)^. Our study recorded that horseshoe breaks and giant retinal tears were significantly associated with the ERM development. Logistic regression analysis further showed that horseshoe breaks and giant retinal tears were significant as well as independent risk factors for the development of ERM. In a previous study, Cacioppo et al.^(3)^ reported that the presence of one or more horseshoe tears was significantly associated with the formation of ERM. The factor underlying these previous findings is that horseshoe breaks arise because of tractional forces. ERM develops via the proliferation of various cells, including retinal pigment epithelium cells, retinal glia, hyalocytes, and other progenitor cells^(13,14)^. It is thus possible that giant retinal breaks allow the cells to flow out easily into the vitreous cavity and migrate freely to the surface of the posterior pole retina. These cells then remain at the vitreoretinal interface and proliferate, thereby promoting the ERM formation.

It is noteworthy that TA staining had a significant preventive effect on the postoperative formation of ERM. In a previous study, Akiyama et al.^(5)^ employed TA staining to visualize ILMs and demonstrated a preventive effect against ERM. These preventive effects of TA may be attributable to both the creation of PVD and the detection of residual vitreous cortex. The attachment of the vitreous to the macula after PVD is also believed to play a certain role in the formation of ERM^(15)^. Even a complete PVD leaves some posterior cortical vitreous attached to the macular area as a result of vitreoschisis or other causes^(16)^. The residual cortex may also help induce the development of ERM by providing a scaffold for cellular proliferation^(15)^. In a previous study, Cho et al.^(17)^ reported evidence that eyes with residual cortex were associated with the development of ERM. The intravitreal injection of TA contributes to the adherence of steroid particles to the vitreous fibers, which can induce a complete PVD and require the removal of the residual cortex attached to the posterior retina, thereby reducing the rate of ERM formation.

Although the restoration of macular anatomy and the improvement of visual outcomes have been previously reported for eyes with idiopathic ERM^(1)^, there have been only limited reports relating to ERM that is secondary to RRD. Previous reports have described only limited improvements in visual acuity following the surgical removal of ERM^(18)^. In this study, we assessed visual acuity in patients undergoing the removal of secondary ERM and noted that the majority of our patients (89.5%) experienced visual improvement and restoration of the foveal profile when examined at the final follow-up visit following the removal of ERM. However, the formation of ERM may limit the functional recovery after ERM peeling.

This study has certain limitations that need to be considered. First, there is the possibility of recruitment bias due to the retrospective nature of our study design, which may have led to an overestimation of the incidence of ERM development considering that cases with favorable visual acuity may have been excluded from our study because of the short follow-up period (<12 months). However, our study indicated that 87.6% of the cases of postoperative ERM were diagnosed within 12 months of the initial RRD repair. Therefore, larger-scale prospective studies are warranted to confirm our results. Second, other factors are known to affect the loss of vision, including silicone oil and the development of postoperative cataracts. During PPV surgery, silicone oil was applied to all eyes because commercialized medical gas was unavailable in the mainland China. Silicone oil was usually removed 3 months after the RRD repair. The exact influence and correlation between silicone oil tamponade and postoperative ERM remain unknown. Silicone oil tamponade has the potential for long-term complications, including cataract. Postoperatively, 4% eyes underwent phacoemulsification with intraocular lens implantation during the silicone oil removal. Nevertheless, most of our patients had a silicone oil tamponade for a rather short period before the removal (94 ± 27 days). Some degree of postoperative cataract formation was recorded in all phakic cases. Third, it is possible that ILM staining with ICG may cause visual impairment postoperatively, hence we cannot completely ignore the possibility of ICG toxicity.

In summary, the overall incidence of postoperative ERM was 28.5%, with 42.7% of the patients undergoing a secondary PPV involving ERM peeling. The presence of horseshoe tears and giant retinal tears were significantly associated with an increased risk of postoperative ERM development. Furthermore, our study demonstrated a significant protective effect of TA staining relating to the ERM development. Most of our patients displayed improved vision following the ERM removal. In contrast, postoperative ERM development led to a reduction in visual acuity when compared with cases without ERM, even after complete surgical removal.
