# ERRATUM

**DOI:** 10.5935/0004-2749.2022-0032E

**Published:** 2025-02-11

**Table:** 

In the article “The development of epiretinal membrane following rhegmatogenous retinal detachment repair: incidence, risk factors, and outcomes” with DOI number: 10.5935/0004-2749.2022-0032, published in the journal Arquivos Brasileiros de Oftalmologia 2022;85(4):370-6, Where it reads: **Conclusão:** Nossa análise demonstra que as lágrimas de ferradura e as lágrimas retinianas gigantes representam fatores de risco para a membrana epirretiniana pós-operatória. Read: **Conclusão:** Nossa análise demonstra que as rasgaduras de ferradura e as rasgaduras retinianas gigantes representam fatores de risco para a membrana epirretiniana pós-operatória.

**Table:** 

No artigo *The development of epiretinal membrane following rhegmatogenous retinal detachment repair*: incidence, risk factors, and outcomes com número de DOI: 10.5935/0004-2749.2022-0032, publicado no periódico Arquivos Brasileiros de Oftalmologia 85.4:370-6, Onde se lia: **Conclusão:** Nossa análise demonstra que as lágrimas de ferradura e as lágrimas retinianas gigantes representam fatores de risco para a membrana epirretiniana pós-operatória. Leia-se: **Conclusão:** Nossa análise demonstra que as rasgaduras de ferradura e as rasgaduras retinianas gigantes representam fatores de risco para a membrana epirretiniana pós-operatória.

